# ACSL4 at the helm of the lipid peroxidation ship: a deep-sea exploration towards ferroptosis

**DOI:** 10.3389/fphar.2025.1594419

**Published:** 2025-08-26

**Authors:** Yulang Jiang, Meng Zhang, Mingyu Sun

**Affiliations:** ^1^ Shuguang Hospital Affiliated to Shanghai University of Traditional Chinese Medicine, Shanghai, China; ^2^ Shanghai University of Traditional Chinese Medicine, Shanghai, China; ^3^ Key Laboratory of Liver and Kidney Diseases, Institute of Liver Diseases, Shuguang Hospital Affiliated to Shanghai University of Traditional Chinese Medicine, Shanghai, China

**Keywords:** ACSL4, lipid peroxidation, ferroptosis, polyunsaturated fatty acids, cancers

## Abstract

Lipid peroxidation stands as a prominent hallmark and a prerequisite for the onset of ferroptosis. Lipid metabolism holds a pivotal role in regulating this process, forming the metabolic foundation for cellular sensitivity to ferroptosis. Studies in lipid metabolomics reveal that the activation of Polyunsaturated fatty acids (PUFA), specifically arachidonic acid and adrenoic acid (AdA), mediated by acyl-CoA synthetase long-chain family member 4 (ACSL4), represents a critical step in generating lipid peroxidation substrates. The expression level or enzymatic activity of ACSL4 emerges as a potential indicator of cellular susceptibility to ferroptosis. Additionally, other members of the ACSL family can indirectly influence the occurrence of ferroptosis by modifying the fatty acid composition of the cell membrane. Given the high expression of ACSL4 in various human tumors, targeting lipid peroxidation with ACSL4 as the focal point may pave a new path in tumor therapy. This article provides a brief overview of the primary structure and function of ACSL4, its role in lipid peroxidation, and summarizes the current advancements in drug development targeting ACSL4 and lipid peroxidation.

## Introduction

Ferroptosis, a unique mode of cell death, relies significantly on the presence of iron ions and is characterized by lipid peroxidation as its hallmark feature (Dixon et al., 2012). This programmed cell death mechanism involves a complex molecular regulatory network, encompassing various signaling pathways and interactions among biological molecules (Stockwell, 2022). During the process of ferroptosis, mitochondria within the cell undergo notable morphological changes: they gradually shrink in size, their membrane density increases, and the cristae structures within the mitochondria decrease (Jiang et al., 2024a). These alterations constitute the specific morphological hallmarks of ferroptosis.

The occurrence of ferroptosis is not an isolated event but is closely intertwined with multiple biological processes such as iron overload, lipid peroxidation, and amino acid metabolism. Lipid metabolism, especially the lipid peroxidation reaction, is considered a prerequisite for triggering ferroptosis. During this process, lipid peroxidation damage leads to the generation of potentially toxic lipid hydroperoxides, which can further exacerbate cell damage and death (Jiang et al., 2024b).

It is worth noting that lipid peroxidation typically occurs in polyunsaturated fatty acids, with arachidonic acid and adrenic acid being the most common. When membrane phospholipids (PUFA-PL) containing these polyunsaturated fatty acids undergo peroxidation, they alter the bilayer structure and geometry of lipids, ultimately resulting in the rupture of the cell membrane and cell death (Chen X. et al., 2021). The delicate balance between non-enzymatic auto-oxidation and enzymatic oxidation reactions of special phospholipids, and the cell’s own antioxidant system, is a critical aspect of phospholipid membrane remodeling in this process (Lee et al., 2021).

Within this intricate regulatory network, ACSL4 plays a crucial role. ACSL4 synthesizes polyunsaturated fatty acid CoA (PUFA-CoA) by adding acetyl-CoA to the carboxyl end of free fatty acids, a key step in the subsequent synthesis of more complex lipid peroxides (Grube et al., 2022). As an indispensable biological factor in ferroptosis, the role of ACSL4 in lipid metabolism is determined by its enzymatic function, which is largely irreplaceable by other ACSL family members. This is the fundamental reason why ACSL4 has a profound impact on the occurrence and development of ferroptosis (Wu et al., 2015). Therefore, ACSL4 is not only regarded as a biomarker of ferroptosis sensitivity but also has the potential to become an important therapeutic target for treating ferroptosis-related diseases in the future.

This article will delve into the specific role and function of ACSL4 in the process of lipid peroxidation-induced ferroptosis, starting from its molecular structure and physiological functions. Simultaneously, utilizing advanced analytical techniques from bioinformatics and transcriptomics, we will further explore the expression of ACSL4 in various types of cancer and its clinical correlation with patient prognosis. Finally, we will summarize and discuss potential small molecule compounds targeting ACSL4 and lipid peroxidation for tumor prevention and treatment, aiming to provide new ideas and directions for future clinical treatment and drug development.

## ACSL family profile

The ACSL family comprises five isoforms in mammals: ACSL1, ACSL3, ACSL4, ACSL5, and ACSL6 (Cheng et al., 2020). Fatty acid activation, regulated by these ACSLs, facilitates both biosynthesis metabolism and catabolism (specifically, fatty acid oxidation) of long-chain lipids. The precise mechanisms that determine which metabolic pathways fatty acids follow remain unknown. Current research indicates that the fate of fatty acids might hinge on the subcellular localization of the ACSLs and their capacity to execute fatty acid metabolism within relatively independent cellular compartment systems. The distribution of various ACSLs differs across subcellular organelles, with ACSL4 predominantly found in the endoplasmic reticulum, mitochondria, plasma membrane, and peroxisome (Long et al., 2025).

The ACSL family exhibits remarkable diversity in genomic organization, chromosomal localization, and functional specialization across isoforms (Table 1). Human ACSL1, mapped to chromosome 4q34.3, contains 21 exons with PPARα-responsive elements in its promoter region, coordinating fatty acid metabolic pathways (Manichaikul et al., 2016). ACSL3, located at 2q36.3, comprises 20 exons and harbors hormone-responsive elements, correlating with its role in prostate cancer progression (Eck et al., 2020). ACSL4, encoded at Xq22.3-q23, contains 14 exons with low interspecies conservation, suggesting evolutionary functional divergence (Chu et al., 2019). ACSL5 (10q25.2) includes 19 exons with gut-specific CDX2 binding sites in its promoter, aligning with intestinal fatty acid absorption (Yamashita et al., 2000). ACSL6, spanning 18 exons at 5q31.2, demonstrates mammalian conservation and brain-specific functional adaptation (Xu W. et al., 2024).

**TABLE 1:** Differences among ACSL family members.

ACSL subtype	Chromosomal location	Number of exons	Key domains/Motifs	Substrate preference	Subcellular localization	Primary functions
ACSL1	4q34.3	21	ATP/AMP binding domain, Fatty acid binding domain, MTS	SFA	Mitochondria, Endoplasmic reticulum	Fatty acid β-oxidation, VLDL assembly
ACSL3	2q36.3	20	ATP/AMP binding domain, Lipid droplet binding domain	MUFA	Lipid droplet surface	Lipid droplet formation, Neutral lipid storage
ACSL4	Xq22.3-q23	14	ATP/AMP binding domain, PUFA binding pocket	PUFA	Peroxisomes	Membrane phospholipid remodeling, Ferroptosis
ACSL5	10q25.2	19	ATP/AMP binding domain, Exogenous fatty acid selectivity domain	Exogenous long-chain fatty acids	Mitochondria	Mitochondrial energy metabolism, Regulation of palmitoylation modifications
ACSL6	5q31.2	18	ATP/AMP binding domain, Brain-specific signal peptide	LCFA	Plasma membrane, Endoplasmic reticulum	Neuronal membrane integrity maintenance, Docosahexaenoic acid metabolism

Despite shared ancestral features—including conserved ATP/AMP-binding domains and fatty acid-binding pockets—structural variations in exon count, regulatory elements, and protein domains underpin tissue-specific expression and metabolic specialization. ACSL1 contains a C-terminal mitochondrial targeting sequence (MTS) and preferentially activates saturated fatty acids for triglyceride synthesis (Schiffman et al., 2018). ACSL3 lacks an N-terminal hydrophobic domain but possesses a lipid droplet-binding motif (LBD) that facilitates neutral lipid storage (Yang Y. et al., 2022). ACSL4 uniquely incorporates a PUFA-specific binding pocket, enabling selective PUFA esterification and ferroptosis modulation (Yang Y. et al., 2022). ACSL5 exhibits exogenous fatty acid selectivity, distinguishing it from endogenously synthesized substrates (Lai et al., 2024). ACSL6 contains an N-terminal brain-specific signal peptide that prioritizes DHA metabolism for neuronal membrane integrity (Di et al., 2024).

Subcellular localization divergence stems from N-terminal sequence variations: ACSL1 localizes to mitochondria-associated membranes and the ER via MTS, participating in β-oxidation and VLDL assembly. ACSL3’s hydrophobic helices mediate lipid droplet association, regulating lipid storage homeostasis. ACSL4 contains a peroxisomal targeting signal (PTS1) for cholesterol transport and membrane phospholipid remodeling. ACSL5’s amphipathic α-helices anchor it to mitochondrial inner membranes, coupling fatty acid activation to energy metabolism. ACSL6 demonstrates dual ER/plasma membrane localization, modulated by palmitoylation-dependent trafficking (Quan et al., 2021).

This structural-functional paradigm—conserved catalytic cores coupled with divergent regulatory/localization domains—enables ACSL isoforms to occupy distinct metabolic niches. While substrate preference (saturated vs polyunsaturated fatty acids) and organelle-specific roles (energy metabolism vs oxidative stress response) differ, all isoforms maintain essential fatty acid activation capacity. These insights provide a structural rationale for developing isoform-selective inhibitors targeting metabolic disorders, cancers, and neurodegenerative diseases (Table 1).

During fatty acid metabolism, these five ACSL isoforms exhibit both overlapping and distinct functional roles. In mitochondria, ACSL1, ACSL4, and ACSL5 primarily contribute to fatty acid synthesis and β-oxidation, while in peroxisomes, ACSL1 and ACSL4 are involved in β-oxidation and alkyl lipid synthesis. The ER, on the other hand, plays a role in β-oxidation and alkyl lipid synthesis, with ACSL1, ACSL3, and ACSL4 in the ER promoting glycolipid synthesis and ω-oxidation. ACSL3, located in lipid droplets, aids in neutral lipid synthesis and lipid droplet formation (Zhang Q. et al., 2023a; Ma Y. et al., 2024a; Klasson et al., 2022; Yen et al., 2017).

**FIGURE 1:**
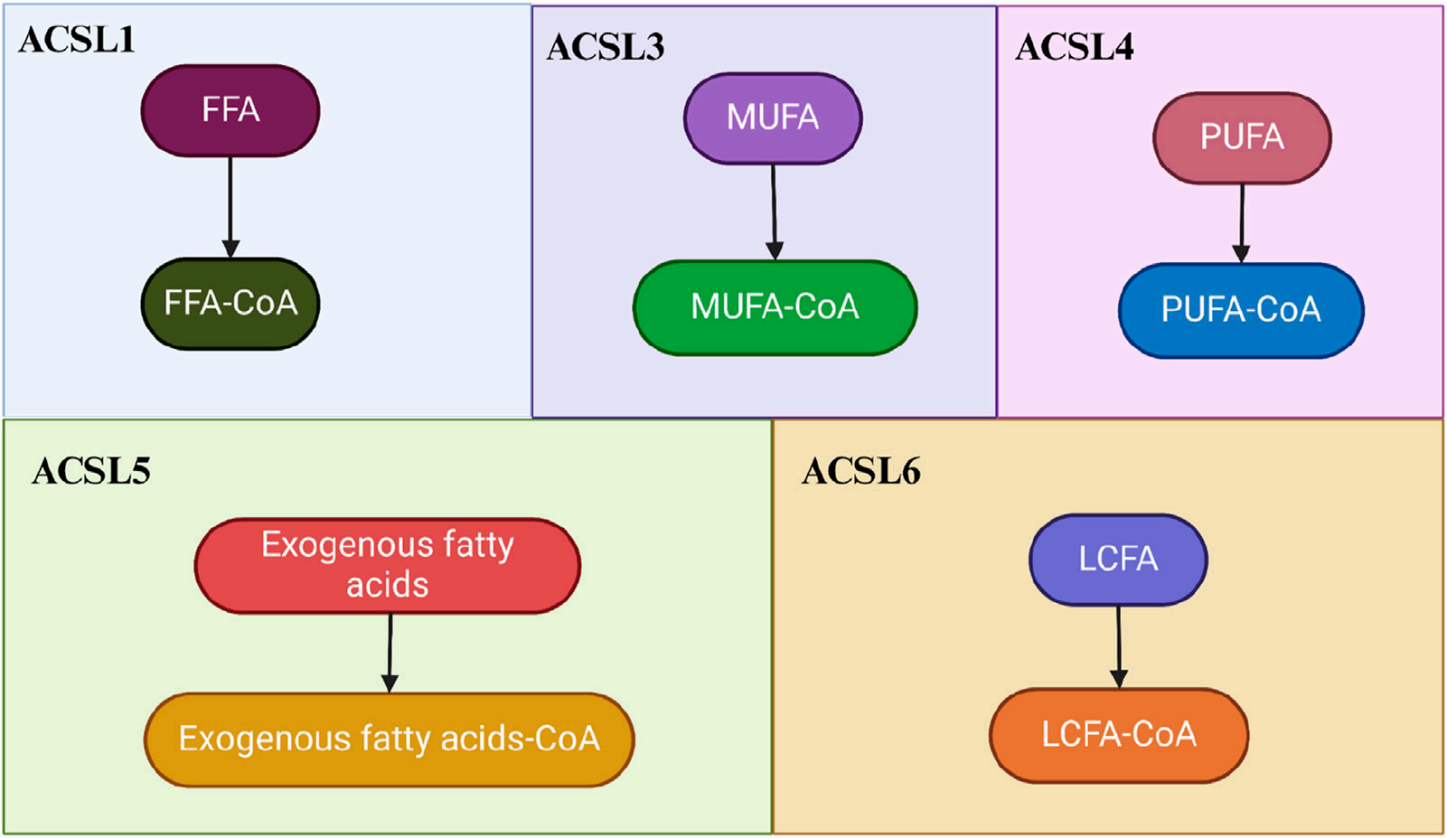
The Roles of ACSLs in Fatty Acid and Lipid Homeostasis. The regulation of ACSLs promotes both the biosynthesis and catabolism of long-chain lipids. In the process of activating fatty acids, the functions of these five isoforms exhibit both overlapping and isoform-specific aspects. Different ACSL isoforms have distinct preferences for substrates: ACSL1 prefers free fatty acids (FFA), ACSL3 favors monounsaturated fatty acids (MUFA), ACSL4 is inclined towards polyunsaturated fatty acids (PUFA), ACSL5 prefers exogenous fatty acids, and ACLS6 preferentially acts on long-chain fatty acids (LCFA).

Furthermore, different ACSL subtypes have preferential substrates. For instance, ACSL1 and ACSL5 demonstrate broad substrate specificity for C12-18 saturated fatty acids and C16-20 unsaturated fatty acids (Yen et al., 2017; Beatty et al., 2021; Chen et al., 2016; Gaisa et al., 2013). Both ACSL3 and ACSL4 activate PUFA, with ACSL3 preferring monounsaturated fatty acids (MUFA) like oleic acid. ACSL4, on the other hand, favors eicosapentaenoic acid, eicosatetraenoic acid (primarily AA and AdA) (Klasson et al., 2022; Migita et al., 2017; Padanad et al., 2016; Feng et al., 2021) (Figure 1).

These findings underscore the significant role of ACSLs in fatty acid metabolism. Whether individual ACSL isoforms direct fatty acids towards anabolism or catabolism may be determined by their subcellular localization and interactions with specific fatty acid transport systems. Notably, PUFA metabolism is a crucial aspect of ferroptosis, and ACSL4 stands out as the only ACSL isoform that plays a significant and direct role in this process (Rochette et al., 2022; Zou et al., 2020). Therefore, exploring the physiological functions of ACSL4 and analyzing changes in its expression could be pivotal for the diagnosis and treatment of ferroptosis-related diseases.

### Expression and prognosis of ACSLs in cancer

An increasing amount of research indicates that various isoforms of ACSL exhibit altered expression patterns in clinical cancers, and certain expressions are closely linked to a poorer prognosis for patients. Table 2 specifically illustrates the modified expressions of different ACSL isoforms across diverse tumors.

**TABLE 2:** Expression and prognosis of different subtypes of the ACSL family in different types of Tumors.

ACSLs	Type of cancer	Expression level	Clinical prognostic relevance
ACSL1	Hepatocellular Carcinoma	up	poor prognosis
	Colorectal Cancer	up	poor prognosis
	Breast Cancer	up	NA
	Leukemia	up	NA
	Lung Cancer	down	NA
	Ovarian Cancer	up	poor prognosis
	Prostate Cancer	up	NA
	Renal Cell Carcinoma	down	poor prognosis
ACSL3	Prostate Cancer	up	NA
	Hepatocellular Carcinoma	up	poor prognosis
	Lung Cancer	up	NA
	Breast Cancer	down	NA
	Renal Cell Carcinoma	up	poor prognosis
	Colorectal Cancer	up	NA
ACSL4	Hepatocellular Carcinoma	up	poor prognosis
	Colorectal Cancer	up	NA
	Breast Cancer	up	poor prognosis
	Prostate Cancer	up	NA
	Gastric Cancer	down	poor prognosis
ACSL5	Glioma	up	NA
	Breast Cancer	down	poor prognosis
	Colorectal Cancer	down	NA
	Bladder Cancer	down	poor prognosis
ACSL6	Breast Cancer	up	favorable prognosis
	Multiple Myeloma	up	poor prognosis

ACSL1 demonstrates high expression in multiple cancers, such as colon, breast, liver, and myeloma, while showing low expression in squamous cell carcinoma of the lung. Notably, colon cancer patients with overexpressed ACSL1 often face a worse prognosis (Liang et al., 2022). Similarly, numerous studies suggest that ACSL4 functions as an oncogene, being overexpressed in colon (Cao et al., 2023), breast (Thomas et al., 2019), liver (Wang et al., 2024), and prostate cancers (Chen J. et al., 2022a), with the exception of gastric cancers where it is overexpressed as well (Zhang et al., 2024). An overexpression of ACSL4 frequently predicts a poorer prognosis for patients with colon and liver cancers. The elevated levels of ACSL4 in these tumors, particularly in colon and liver cancers, could potentially enable targeted induction of ferroptosis in tumor tissues (Li and Li, 2020).

Conversely, ACSL5 exhibits an opposing trend in cancer expression. Apart from being overexpressed in gliomas, ACSL5 demonstrates low expression in various other cancers, including colon and breast (Quan et al., 2021). This contrasts with the upregulated expression of ACSL1 and ACSL4 in these cancers. Additionally, ACSL5 expressions are downregulated in bladder cancer. It is worth noting that in breast cancer, lower ACSL5 expression correlates with a poorer patient prognosis (Rossi Sebastiano and Konstantinidou, 2019).

The role of ACSL3 in cancer is multifaceted. It is overexpressed in lung cancer, prostate cancer, and estrogen receptor-negative breast cancer. Among these, an upregulation of ACSL3 predicts a worse prognosis for prostate cancer patients (Klasson et al., 2022). However, conflicting results have emerged from studies on prostate and breast cancers (Xie et al., 2022a), where ACSL3 expression is reduced in metastatic prostate cancer (Migita et al., 2017). The complete deletion of ACSL3 has been linked to an elevated risk of breast cancer (Tan et al., 2024) recurrence and distant metastasis in triple-negative breast cancer following chemotherapy (WrighT et al., 2017). These contradictory findings suggest that ACSL3 might play varying roles depending on the cancer stage (Shi et al., 2023).

Regarding ACSL6, its role in cancer is less frequently documented. In triple-negative breast cancer, ACSL6 expression significantly increases, and patients with high ACSL6 expression tend to have a better survival prognosis (Hua et al., 2024). This might be associated with a positive correlation between ACSL6 and memory CD4 T cell infiltration, along with a negative correlation with macrophages and resting dendritic cells. Meanwhile, a case report has identified an ETV6-ACSL6 gene fusion in chronic leukemia, rendering cancer cells resistant to tyrosine kinase inhibitors. Nevertheless, the significance of ACSL6 as a diagnostic and prognostic marker in tumor therapy still requires further clinical trial data to support its validity (Wu et al., 2020).

In summary, ACSL1 and ACSL4 are suspected to contribute to tumor progression in most of the tumor types examined, whereas ACSL5 appears to hinder tumor growth. Conversely, the function of ACSL3 in cancer seems to hinge on the specific type and stage of the disease. Furthermore, the elevated expression of ACSL4 observed in certain cancer tissues suggests that it could serve as a potential molecular target for triggering ferroptosis.

### Structure of ACSL4 and its function

The ACSL4 protein, consisting of approximately 711 amino acid residues, boasts a molecular weight of roughly 80 kDa and an isoelectric point of 8.66. By employing cryo-electron microscopy, we can discern the four primary components of the ACSL4 protein: the N-terminal membrane-bound domain, the transmembrane domain, the adenylate-binding domain, and the C-terminal catalytic domain (Wu et al., 2024).

Within the ACSL4 protein, the N-terminal region leads the structure and comprises two somewhat unstable subregions, N1 and N2, predominantly made up of leucine-rich segments. Despite our incomplete understanding of this region’s precise function, it is postulated to significantly contribute to the facilitation of the fatty acid acylation reaction. This process involves the conjugation of fatty acids with coenzyme A, yielding acyl coenzyme A. These compounds serve as an energy source and constitute a crucial aspect of intracellular fatty acid metabolism. Additionally, this region potentially contributes to protein localization (Jia et al., 2023).

Meanwhile, transmembrane polypeptides primarily function in penetrating the cell membrane, bridging the N-terminal and C-terminal domains, and harbor a designated pocket for binding long-chain fatty acids.

Moving on, the adenosine monophosphate (AMP) linker region emerges as the third pivotal component of ACSL4. This adenosine-related domain primarily connects adenosine triphosphate (ATP) and CoA molecules. It comprises two substructures: a nucleotide-binding segment and a CoA-binding segment, interconnected by a cantilever peptide, collectively forming the AMP linker region (Xue et al., 2023; Kong et al., 2023).

Lastly, the C-terminal domain emerges as a critical aspect of the ACSL4 protein, catalyzing the acylation reaction and forming the catalytic hub of ACSL4. It is worth noting that the structure of ACSL4 bears remarkable similarity to other ACSL proteins. Their catalytic centers all encompass a conserved acylase kinetic triad: lysine (Lys)-aspartate (Asp)-cysteine (Cys). Within this triad, Lys and Asp facilitate protonation and deprotonation during the catalytic acylation, whereas Cys serves as the pivotal site for fatty acid binding to CoA (Jiang and Sun, 2024).

## Role of ACSL4 in lipid peroxidation and ferroptosis

### Regulation of PUFA by ACSL4

**FIGURE 2:**
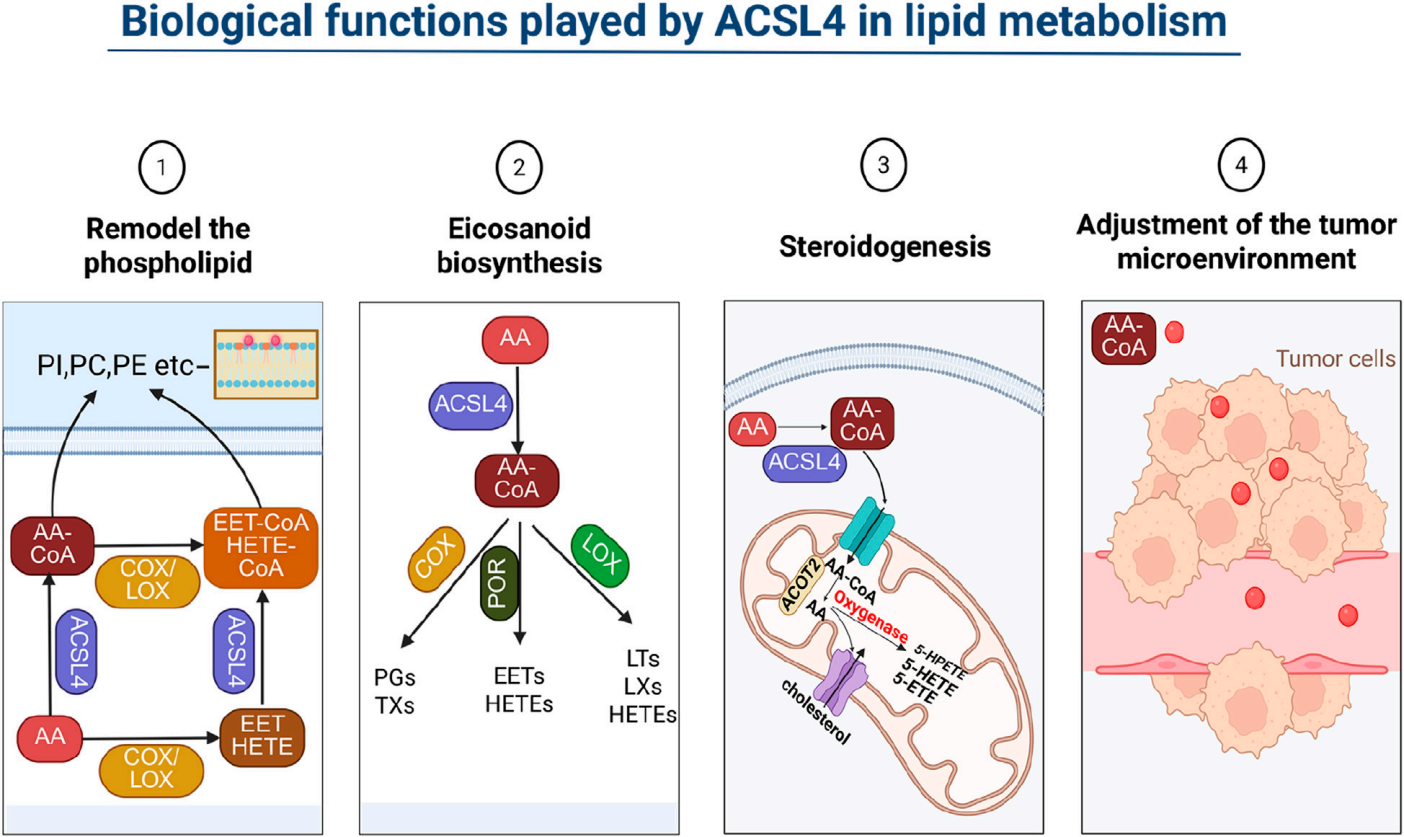
Biological functions played by ACSL4 in lipid metabolism. ACSL4 influences cell membrane lipid composition by impacting lipid metabolism, which subsequently affects cell permeability. By adding CoA to PUFA, ACSL4 participates in eicosanoid acid and steroid metabolism, thereby exerting an impact on the immune microenvironment of tumor cells.

The occurrence of ferroptosis, a form of cell death, is contingent on the extent of lipid peroxidation, which in turn, is influenced by the synthesis and metabolism of polyunsaturated fatty acids (PUFAs). The regulation of PUFA biosynthesis involves a intricate process. To illustrate, in the case of eicosatetraenoic acid, numerous arachidonic acid (AA) metabolizing enzymes, including phospholipase A2 (PLA2), cyclooxygenase (COX), lipoxygenase (LOX), and cytochrome P450 (CYP), play a role in this regulation (Zhou et al., 2024). Typically, either COX or LOX triggers the release of AA, and the oxidized AA is then transformed into various eicosanoids through the catalysis of distinct eicosanoid synthases. Cytochrome P450 monooxygenase catalyzes the synthesis of epoxyeicosatrienoic acid (EET) and hydroxyeicosatetraenoic acid (HETE). Both EET and HETE can be activated by ACSL4, leading to the generation of EET-CoA and HETE-CoA. These oxidized acyl coenzymes undergo esterification with phospholipids, thereby impacting the formation of membrane lipid bilayers (Yang D. et al., 2022b; Zeeshan et al., 2016; Krajka-Kuźniak and Baer-Dubowska, 2003) (Figure 2).

Studies in lipid metabolomics have elucidated the role of ACSL4 in regulating the formation of phospholipids from AA. Additionally, these studies have revealed that the amount of linoleic acid (LA) involved in phospholipid synthesis escalates with ACSL4 deficiency (Nava Lauson et al., 2023). Crucially, during ferroptosis, AA and AdA are activated by ACSL4, which catalyzes the reaction between AA and CoA to produce the intermediate AA-CoA. This reaction necessitates the consumption of adenosine triphosphate (ATP). Elongase5 acts on AA-CoA, adding two carbon atoms to yield AdA-CoA. Both AA-CoA and AdA-CoA are esterified by LPCAT3 to form AA-PE and AdA-PE, respectively (Gupta et al., 2023; Cui et al., 2023). Subsequently, these specific unsaturated phospholipids can undergo oxidation to generate lipid hydroperoxides (PE-AA-OOH and PE-AdA-OOH) either through autoxidation or by the action of 15-lipoxygenase (15-LOX), thereby promoting ferroptosis (Singh and Rao, 2019; Aliabadi et al., 2023). If the formation of AA-PE is obstructed, the process of ferroptosis is impacted. GPx4 reduces these lipid peroxides to their corresponding lipid alcohols. Furthermore, antioxidant molecules, such as reduced coenzyme Q10 (CoQ10), possess the ability to hinder the oxidation process of lipid peroxides (Samovich et al., 2024).

Although the concentration of AA in the cell membrane is very low, at the nanomolar level, ACSL4 exhibits high specificity and affinity for AA. As a result, ACSL4 preferentially utilizes AA for phospholipid synthesis. Therefore, the level of ACSL4 directly determines the concentration of specific lipid peroxides, indirectly influencing the cell’s sensitivity to ferroptosis. PEBP1 is another crucial protein involved in lipid peroxidation. By forming a complex with 15-LOX, PEBP1 aids 15-LOX in recognizing PE-AA and PE-AdA, thereby promoting their peroxidation and ultimately inducing ferroptosis in cells (Wenzel et al., 2017).

The catalytic selectivity of ACSL4 for exogenous fatty acids varies in different tissue cells, due to the differential expression of ACSL4 and other metabolic enzymes caused by different cell types, as well as the varying fatty acid compositions within different cells. For instance, the absence of ACSL4 in adipocytes reduces the incorporation of AA into phospholipids and the AA pool, and lowers the level of 4-hydroxynonenal, a product of AA lipid peroxidation. Overexpression of ACSL4 in human arterial smooth muscle cells significantly promotes the synthesis of phosphatidylethanolamine (PE) and phosphatidylinositol (PI) from exogenous AA. Similarly, overexpression of ACSL4 in fibroblast-like COS-7 cells facilitate the formation of PE from exogenous AA and oleic acid (OA) (Amtul et al., 2011). In COS-7 cells, exogenous AA also participates in the synthesis of phosphatidylcholine (PC), while steroidogenic cells regulate the release of AA through the acyl-CoA thioesterase 2 (ACOT2) pathways (Moffat et al., 2014). Subsequently, ACSL4 catalyzes the conversion of intracellular free AA to AA-CoA, which is then provided to ACOT2 for the release of AA in mitochondria. The released AA is metabolized through the lipoxygenase pathway, inducing the steroidogenic acute regulatory protein (StAR). The StAR protein is a rate-limiting enzyme in steroid hormone biosynthesis, controlling the transport of cholesterol into the inner mitochondrial membrane. The expression of StAR is also feedback-regulated by AA or its metabolites in these cells (Hou et al., 2024).

Although the peroxidation of both PE-AA and PE-AdA can trigger ferroptosis in cells, the intracellular concentration of PE-AA is significantly higher than that of PE-AdA. As for whether the two have a synergistic effect, there is currently no definitive conclusion. PE, as a phospholipid related to ferroptosis lethal signaling, may be due to its predominant presence in the inner leaflet of the cell membrane phospholipid bilayer, which facilitates the action of lipid oxygenases (Luo et al., 2023). On the other hand, PC, which exists in the outer leaflet of the lipid bilayer, is not conducive to its oxidation by lipid oxygenases. Additionally, phospholipids are amphiphilic molecules, and their assembly form is controlled by the critical packing parameter (CPP). Most phospholipids typically assemble into lipid bilayers, while PE-AA and PE-AdA can assemble into hexagonal phases. This behavior also facilitates the binding of lipid oxygenases, providing conditions for subsequent enzymatic catalytic reactions. The simultaneous delivery of ferroptosis inducers and lipid oxygenase inhibitors can limit the peroxidation of PE-AA and PE-AdA, thereby effectively inhibiting the occurrence of ferroptosis (Johansson et al., 2023). Cytochrome P450 oxidoreductase (POR) is another enzyme, besides 15-LOX, that can catalyze the lipid peroxidation of PE-AA and PE-AdA, further enriching the theory of ferroptosis lipid peroxidation (Yan et al., 2021; Liu et al., 2022).

**FIGURE 3:**
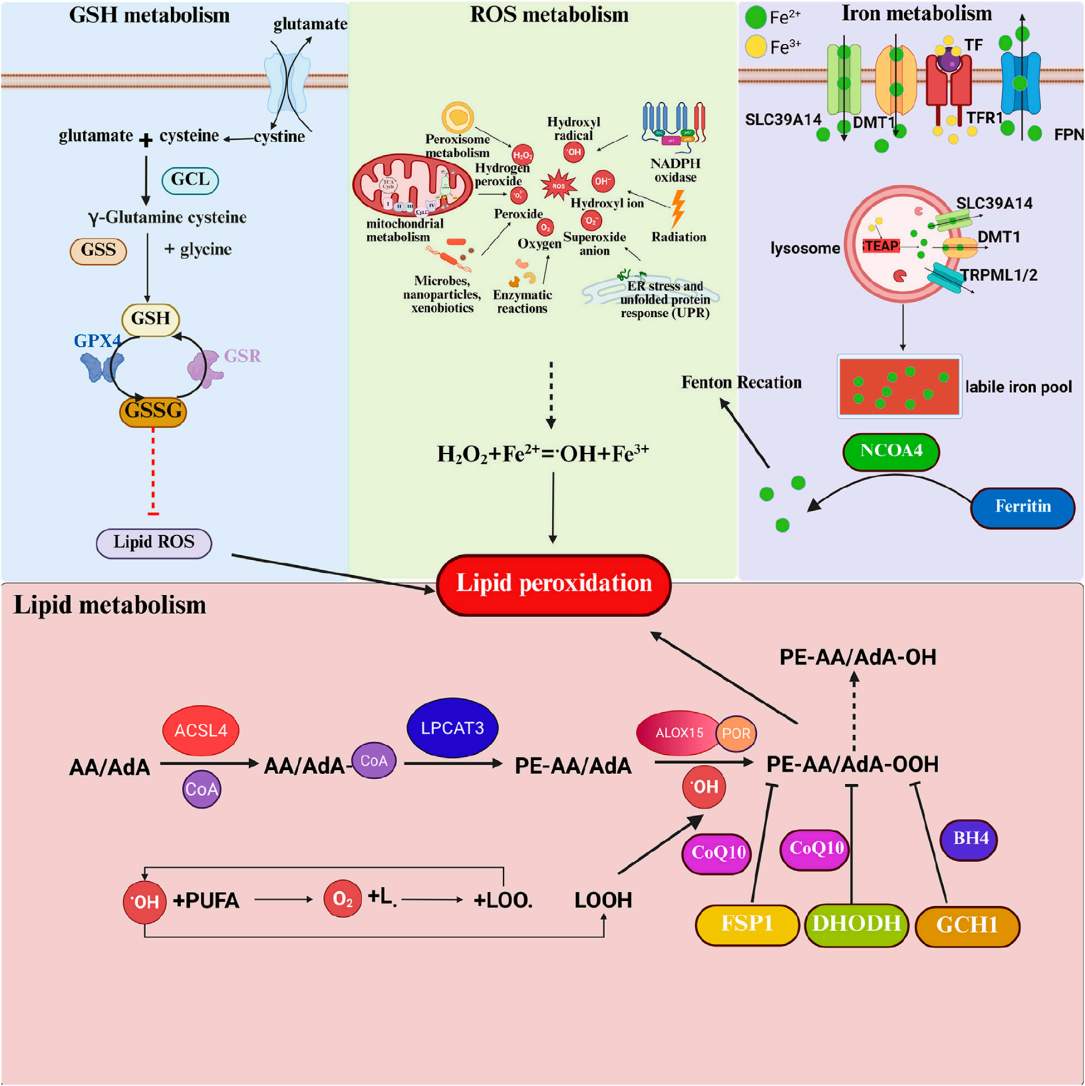
Regulation mechanism of ACSL4 and lipid peroxidation in ferroptosis. Ferroptosis involves the metabolism of GSH, ROS, iron ions, and lipids. As a crucial intracellular antioxidant, GSH plays a pivotal role in the process of ferroptosis. The synthesis of GSH relies on System Xc-, which is responsible for transporting extracellular cystine into the cell, where it is rapidly reduced to cysteine. Inside the cell, cysteine, along with glutamic acid and glycine, undergoes a two-step enzymatic reaction to produce GSH. GSH serves as a cofactor for GPX4, reducing intracellular lipid peroxides. The core mechanism of ferroptosis is the imbalance of ROS. ROS are primarily generated by cellular organelles such as mitochondria and peroxisomes during oxidative phosphorylation, fatty acid beta-oxidation, and other processes. Iron ions, particularly ferrous ions (Fe^2+^), catalyze the production of highly reactive hydroxyl radicals (·OH) from hydrogen peroxide (H_2_O_2_) through the Fenton reaction, further exacerbating ROS generation. Iron ions typically enter cells in the form of ferric ions (Fe^3+^) bound to transferrin, mediated by transferrin receptor (TfR1) endocytosis. Inside the cell, Fe^3+^ is reduced to Fe^2+^, and the accumulation of iron ions is one of the key factors in the process of ferroptosis. Excess Fe^2+^ forms a labile iron pool (LIP) within the cell, participating in the Fenton reaction and ultimately triggering ferroptosis. The primary source of lipid peroxides is unsaturated fatty acids, especially arachidonic acid (AA) and adrenic acid (AdA). They are first activated by acyl-CoA synthetase long-chain family member 4 (ACSL4) to form AA/AdA-CoA. The activated AA/AdA-CoA further reacts with phosphatidylcholine under the catalysis of Lys phosphatidylcholine acyltransferase 3 (LPCAT3) to generate AA/AdA-PE. Finally, AA/AdA-PE undergoes lipid peroxidation catalyzed by lipoxygenases (LOXs), producing lipid hydroperoxides that disrupt membrane structure and ultimately lead to ferroptosis.

The mechanism by which specific lipid peroxides and peroxidative free radicals induce ferroptosis in cells involves several aspects. Firstly, the accumulation of lipid peroxides in cell membranes and subcellular organelle membranes can alter membrane fluidity, permeability, and integrity. Secondly, lipid peroxides can damage biological macromolecules such as proteins embedded in the bilayer phospholipid membrane. Finally, degradation byproducts of lipid peroxides (such as malondialdehyde and 4-hydroxynonenal) can harm various important biological macromolecules like proteins and nucleic acids within the cell (Zhang X. et al., 2023b). The lipid peroxidation process that induces ferroptosis can occur in the plasma membrane, as well as the outer membranes of mitochondria, endoplasmic reticulum, and lipid droplets. Among these, mitochondria, as one of the main sources of endogenous reactive oxygen species, are very sensitive to lipid peroxidation. The accumulation of PE-AA-OOH and PE-AdA-OOH in the outer mitochondrial membrane can lead to increased membrane permeability, mitochondrial swelling, and ultimately outer membrane rupture (Liu et al., 2023). However, the mechanism by which specific lipid peroxides trigger mitochondrial fragmentation remains unclear (Figure 3).

### Initiation, propagation and termination stages of lipid peroxidation

Phospholipids and other lipid molecules containing unsaturated double bonds are prone to autoxidation in the presence of oxygen, which is essentially a free radical chain reaction. This process often begins with the cleavage of the peroxide O-O bond induced by divalent iron or similar low-valent metal ions, producing a highly reactive hydroxyl radical (HO.) This radical then proceeds to extract hydrogen from the unsaturated lipid, giving rise to a lipid radical (L.), effectively triggering the free radical chain reaction.

As this reaction unfolds, L. interacts with molecular oxygen, leading to the formation of lipid peroxyl radical (LOO.) This LOO. radical subsequently targets another unsaturated lipid, resulting in the generation of L. and lipid peroxyl (LOOH), thus entering the amplification phase of the free radical reaction. Additionally, LOOH can also be derived from the enzymatic catalysis of unsaturated lipids by lipoxygenases (Homma et al., 2021; Xu J. et al., 2024b).

The speed of this chain reaction is closely linked to the degree of unsaturation within the lipid molecule; a higher degree of unsaturation leads to a greater amplification rate constant, intensifying the chain reaction. Finally, the reaction terminates when two molecules of LOO. interact (Xu J. et al., 2024b; Su et al., 2019).

**FIGURE 4:**
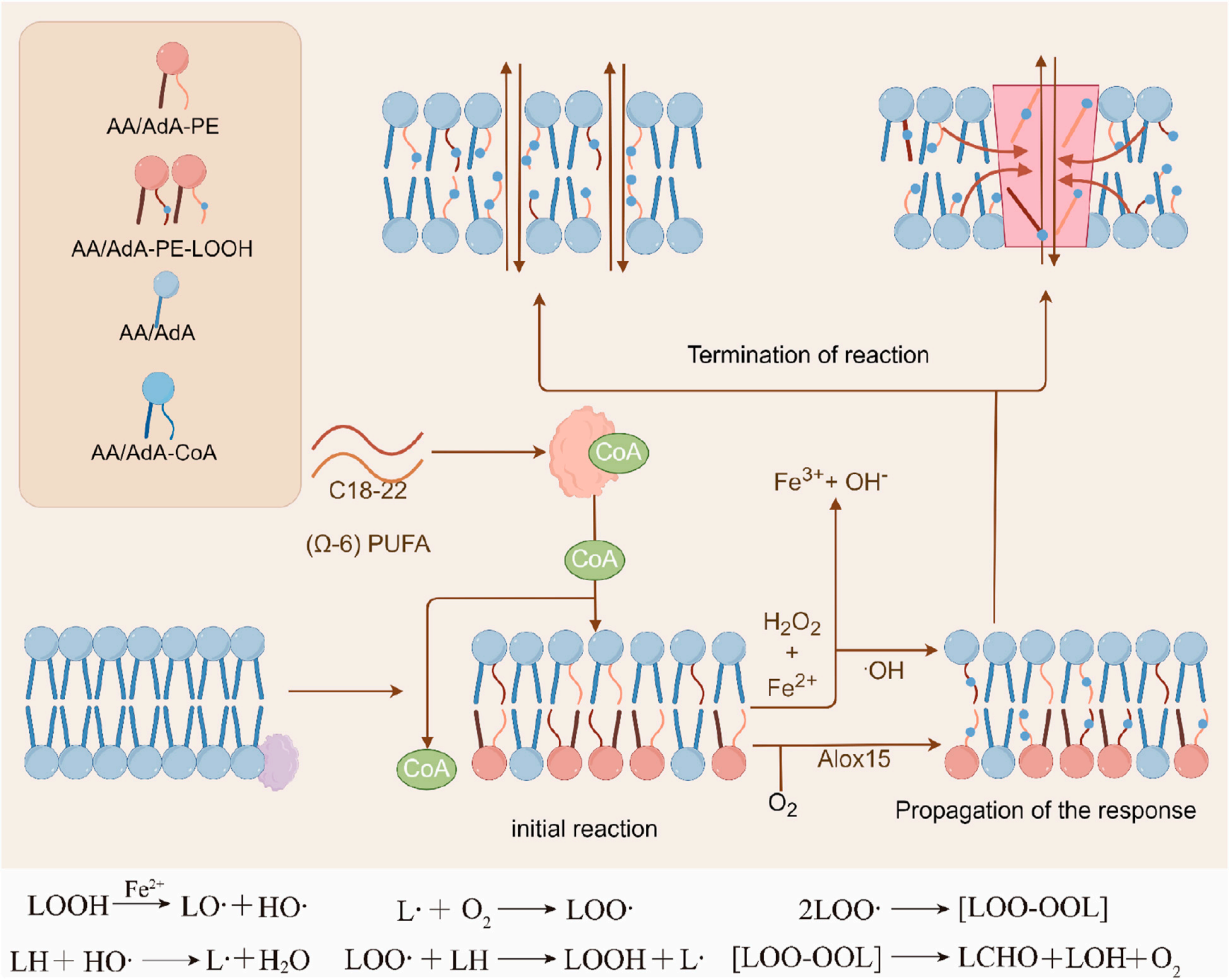
Three stages of lipid peroxidation. Phospholipids or other lipid molecules containing unsaturated double bonds can undergo lipid peroxidation reactions, which mainly include the initiation, propagation, and termination stages. Initially, divalent iron can cause the cleavage of the O-O bond to form highly reactive hydroxyl radicals (HO·), which further abstract hydrogen from unsaturated lipids to generate lipid radicals (L·), initiating a free radical chain reaction. Subsequently, L reacts with molecular oxygen to form lipid peroxyl radicals (LOO·), which further attack another unsaturated lipid molecule to generate L· and lipid hydroperoxides (LOOH), entering the stage of free radical propagation reaction. The propagation rate of the free radical chain reaction is usually related to the degree of unsaturation of the lipid molecules. In the termination stage, antioxidants can provide a hydrogen atom to the hydroperoxide, forming non-radical products.

During the initiation stage, phospholipids with polyunsaturated fatty acid side chains (PL-PUFA) are susceptible to attack by free radicals (non-enzymatic reactions) or can undergo enzymatic reactions catalyzed by oxidation-related enzymes. In the termination phase, antioxidants like vitamin E contribute by providing a hydrogen atom to lipid hydroperoxides, thereby forming a stable, non-free radical product (Miotto et al., 2020) (Figure 4).

Given that the propagation of phospholipid oxidation is a non-enzymatic, stochastic process, regulating the initiation of phospholipid oxidation becomes a crucial aspect in managing lipid peroxidation.

### ACSL4-independent ferroptosis

Although ACSL4 is a key enzyme regulating the esterification of polyunsaturated fatty acid phospholipids and significantly promotes ferroptosis induced by GPX4 inhibitors such as RSL3, its dependency is significantly reduced in ferroptosis induced by cystine depletion or system Xc-inhibitor erastin. Studies have shown that in ferroptosis screens triggered by GPX4 inhibitors, 88% (14 out of 16) of the screening results identified ACSL4 as an inhibitory factor (whose inactivation can inhibit ferroptosis), but in screens induced by erastin or cystine starvation, only 12% (1 out of 8) of the screening results identified ACSL4 as an inhibitory factor (Lee and Gan, 2022). At the same time, through CRISPR-Cas9 and pharmacological inhibition experiments, it has been confirmed that ACSL4-deficient cells are far more resistant to GPX4 inhibitor-induced ferroptosis than to erastin-induced ferroptosis. This indicates that ACSL4 plays a more critical role in mediating GPX4 inhibition-induced ferroptosis than in SLC7A11 inhibition-induced ferroptosis (Wang X. et al., 2022a). Some studies have also shown that when ACSL4 is absent, LPCAT3 can directly insert free PUFAs (such as AA, AdA) into PE or phosphatidylinositol (PI) to generate oxidizable PUFA-PLs, driving lipid peroxidation. Unesterified free PUFAs can also be oxidized in lipid droplets or plasma membranes, accumulating lipid peroxides through non-enzymatic reaction. In p53-mediated ferroptosis, ALOX12, rather than ACSL4, is the main executor, indicating the existence of heterogeneous ferroptosis pathways in different disease contexts. These findings challenge the current view that ACSL4 is a universal regulatory factor of ferroptosis (Magtanong et al., 2022).

The existence of such heterogeneous ferroptosis pathway networks provides a molecular target library for precision intervention. For example, prioritizing the targeting of ALOX12 in p53-mutated tumors and designing ACSL4-LPCAT3 dual-target inhibitors in GPX4-low expressing cancers may break through the efficacy bottleneck of existing ferroptosis inducers, pushing metabolic targeted therapy into a new era of “signal pathway decoding” (Chu et al., 2019).

## Impact of lipid peroxidation damage and ferroptosis on membrane-bound organelles

When lipid peroxidation homeostasis is disrupted, cells undergo death. During the late stage of ferroptosis, cells exhibit swelling and compromised membrane integrity. *In vitro* studies have revealed that cells undergoing ferroptosis can induce ferroptosis in neighboring cells, and this propagative effect relies on the catalysis of iron ions and lipid peroxidation. Specifically, lipid peroxidation disrupts ion concentration gradients, reduces membrane fluidity, impedes lateral diffusion, and increases membrane permeability. Ferroptosis is particularly prone to occur in cells with deformable membranes. The presence of PUFAs in glycerophospholipids reduces membrane bending stiffness, thereby facilitating membrane deformation (Hu L. et al., 2023a).

### Mitochondria

Mitochondria serve as a central hub for cellular metabolism, signal transduction, and the regulation of death pathways. Notably, mitochondrial morphological alterations and functional contributions during ferroptosis progression are more pronounced compared to other organelles. While their primary role in energy production is critical for cellular fate decisions, mitochondria also function as the main site for fatty acid, amino acid, iron, and carbon metabolism. The mitochondrial respiratory chain has long been recognized as a primary source of ROS, including superoxide anions. Dysregulation of mitochondrial function and metabolic reprogramming—triggered by intracellular or extracellular cues—profoundly influence cell survival outcomes (Deng et al., 2025).

Accumulating evidence indicates that diverse metabolic pathways, including iron, lipid, and amino acid metabolism, converge to induce ferroptosis. Recent studies have positioned mitochondria as a key executor organelle in RCD processes, including ferroptosis, where they mediate the release or sequestration of specific pro-death factors. This regulatory nexus between RCD and mitochondrial integrity is predominantly governed by MPTP opening and alterations in outer mitochondrial membrane (OMM) permeability (Song et al., 2025).

A distinctive feature differentiating ferroptosis from other RCD modalities lies in its characteristic mitochondrial morphological changes. Post-ferroptotic mitochondria exhibit reduced volume, increased matrix density, elevated membrane potential, cristae depletion, and OMM rupture—phenotypes that contrast sharply with apoptotic or necrotic morphological patterns (Al Amir Dache and Thierry, 2023). Time-dependent mitochondrial membrane disruption has been observed in MEFs undergoing RSL3-induced ferroptosis. Notably, pharmacological inhibition or genetic ablation of ACSL4—a critical executor of ferroptotic machinery—significantly enhances mitochondrial resistance to RSL3-triggered OMM rupture (Annesley and Fisher, 2019). These findings collectively underscore mitochondria as pivotal effectors in ferroptosis execution.

### Endoplasmic reticulum

ER stress, induced under diverse pathological conditions, is intricately linked to cell death mechanisms. ER stress response genes modulate apoptotic or autophagic pathways, and the ferroptosis inducer erastin has been shown to activate ER stress while upregulating pro-stress genes (Rozpedek et al., 2016). The Eif2α-ATF4 axis serves as the primary signaling pathway mediating ferroptotic activation within the ER. Among ATF4 downstream effectors, CHAC1 facilitates GSH depletion and promotes ferroptosis progression. PUMA, another ATF4 target gene, is also elevated during ER stress induced by the ferroptosis agonist artemisinin (ART). However, PUMA activation under ferroptotic stimuli paradoxically triggers caspase-dependent apoptotic cell death rather than ferroptosis, leaving the functional role of ER-resident PUMA in ferroptosis execution unresolved (Hao et al., 2021).

Furthermore, compelling evidence indicates that ER stress induced by ferroptotic agents cannot be alleviated by lipid peroxidation inhibitors Fer-1 or Lip-1. This observation underscores the current lack of direct evidence linking ER stress response genes to ferroptosis regulation. From a lipogenic perspective, the ER constitutes the initial intracellular site for lipid synthesis. Lipid peroxidation initiates within the ER lumen before propagating to other cellular compartments. Within the ER, the lipoxygenase 15-LOX oxidizes AA-CoA to generate arachidonoyl-CoA hydroperoxide AA-OOH-CoA. Notably, AA-OOH-CoA does not contribute to RSL3-induced ferroptosis, suggesting that peroxidation of phosphatidylethanolamine species containing arachidonic acid (C20:4) and adrenic acid (C22:4) directly drives ferroptotic demise, rather than cytoplasmic oxidation of free polyunsaturated fatty acids (Xie et al., 2023). Despite these insights, the precise initiation site of lipid peroxidation during ferroptosis remains elusive and warrants further investigation.

### Lysosomes

Lysosomes emerge as critical participants in ferroptotic pathways. Studies reveal that lysosomal-derived ROS constitute the primary source of oxidative stress in HT1080 cells undergoing erastin- or RSL3-induced ferroptosis (Qi et al., 2024). Pharmacological inhibition of lysosomal activity effectively suppresses both lysosomal ROS production and ferroptosis-associated oxidative bursts. Through modulation of intracellular iron homeostasis—either by attenuating transferrin-mediated iron import or impairing ferritin autophagic degradation—lysosomes regulate iron availability for ferroptotic execution. Notably, inhibition of lysosomal cathepsin B (a cysteine protease) activity reduces cellular sensitivity to erastin-triggered ferroptosis (Wei et al., 2020). In human PDAC models, STAT3-mediated regulation of cathepsin B expression further implies lysosomal function in ferroptosis progression.

Recent investigations propose ferroptosis as an autophagy-dependent form of RCD, with lysosomes serving as key organelles for autophagic degradation of protein aggregates. Supporting this paradigm, genetic ablation of autophagy-related genes (ATG13/ATG3) significantly attenuates cysteine deprivation-induced ferroptosis. Autophagy may promote ROS generation and lipid peroxidation accumulation through NCOA4-mediated ferritinophagy—a selective autophagy process that degrades ferritin to release labile iron pools (Huang et al., 2024). Consistent with this mechanism, NCOA4 knockout diminishes ferritinophagy, reduces cytosolic free iron levels, and suppresses ferroptosis by limiting ROS accumulation. However, direct causality between autophagy activation and ferroptosis execution remains unresolved (Zhu et al., 2023).

Controversially, emerging evidence challenges the lysosome’s centrality in ferroptosis. Notably, ferrostatins—classical ferroptosis inhibitors—localize to lysosomes, yet structural modifications that reduce their lysosomal accumulation paradoxically enhance anti-ferroptotic efficacy. This observation raises questions about the lysosome’s obligatory role *versus* its potential compensatory involvement in ferroptotic pathways.

### Golgi apparatus

Golgi apparatus stress contributes to ferroptosis regulation in human cells. Compounds perturbing Golgi function—including AMF-26, BFA, GCA, and AG1478/yrphostin—induce ferroptotic cell death. Conversely, co-treatment with various ferroptosis-modulating agents attenuates Golgi disruption, preserving organelle morphology and functional integrity (Ignashkova et al., 2017). During exposure to Golgi stressors, ferroptosis inhibitors demonstrate protective effects by preventing Golgi fragmentation and suppressing aberrant protein secretion. Notably, sublethal erastin concentrations mitigate lipid peroxidation triggered by Golgi stress, suggesting a compensatory role for the transsulfuration pathway in maintaining cysteine availability under oxidative stress. Pharmacological inhibition of transsulfuration abrogates the cytoprotective synergy observed between low dose erastin and Golgi stressors, indicating functional interplay between these pathways (Ohashi et al., 2012).

Collectively, these findings establish the Golgi apparatus as a critical regulator of cellular redox homeostasis and ferroptosis susceptibility. The organelle’s involvement extends beyond mere structural disruption, encompassing metabolic reprogramming that modulates ferroptotic sensitivity. This dual regulatory capacity positions the Golgi as both a stress sensor and effector in ferroptosis pathways, with implications for therapeutic strategies targeting redox-vulnerable malignancies.

### Nuclear

Contrary to conventional understanding that nuclear alterations are inconspicuous during ferroptosis with preserved chromatin integrity, TEM analyses reveal distinct nuclear morphological changes. In erastin-treated HT1080 and A549 cells, TEM imaging demonstrated progressive cytoplasmic vacuolization accompanied by electron-lucent cytoplasmic transformation correlated with ferroptotic severity. Paradoxically, nuclei also exhibited electron lucency—a phenomenon sharply contrasting with necrotic cells—while maintaining intact perinuclear spaces and remaining embedded within expanded cytoplasm rather than extruding into the extracellular space. Notably, plasma membrane integrity remained compromised but structurally intact during late-stage ferroptosis (Liu et al., 2022).

The observed nuclear electron lucency in erastin-treated cells prompted investigation into nuclear protein release. Immunodetection of the nuclear localization protein HMGB1 via Western blot and immunofluorescence staining revealed progressive translocation from the nucleus to the cytoplasm and ultimately to the extracellular space during ferroptosis progression (Chen R. et al., 2022b). These findings challenge the traditional paradigm by suggesting that nuclear membrane disruption precedes plasma membrane rupture in ferroptosis.

## The role of ACSL4 in other lipid peroxidation-induced diseases

Ferroptosis, a form of regulated cell death characterized by lipid peroxidation, is implicated in diverse physiological and pathological processes across multiple tissues and organs. Studies demonstrate its significant role in the pathogenesis of neurodegenerative disorders, including Alzheimer’s disease, Parkinson’s disease, multiple sclerosis, and amyotrophic lateral sclerosis. In organ transplantation, ferroptosis is a key mechanism underlying tissue damage following ischemia-reperfusion injury (IRI) in the heart, kidney, liver, and lungs. Furthermore, this process is closely associated with the pathological progression of acute kidney injury (AKI), chronic kidney disease (CKD), atherosclerosis, and heart failure. Per your suggestion, we have expanded the discussion on the role of ACSL4-mediated lipid peroxidation in ferroptosis-related diseases, as detailed below:

Ferroptosis represents a cell death pathway relevant to numerous conditions, including cancer, cardiovascular diseases, and degenerative brain disorders, making it a focus of extensive research. Within this context, ACSL4 has been widely reported as a biomarker of ferroptosis sensitivity. While ACSL4 promotes ferroptosis to eliminate tumor cells, its upregulation also suggests a pathogenic role in ferroptosis-associated diseases. As noted, ferroptosis significantly contributes to neurodegenerative disorders and is a critical mediator of IRI in transplanted organs (heart, kidney, liver, lungs) (Li et al., 2023). It is also mechanistically linked to AKI, CKD, atherosclerosis, and heart failure.

Studies utilizing ferroptosis inhibitors in animal models demonstrate protection against ischemic injury in the liver, kidney, brain, and heart. A study on myocardial IRI found that while prolonged ischemia exacerbated injury without altering key ferroptosis markers, reperfusion triggered a significant increase in ACSL4, iron, and MDA levels, concomitant with GPx4 downregulation. Pretreatment with the iron chelator DFO showed no protective effect during ischemia alone in rat hearts. However, DFO administered during IRI reduced ACSL4 expression, suppressed ferroptosis, and markedly attenuated myocardial injury. These findings indicate that ferroptosis predominantly occurs during the reperfusion phase rather than ischemia, and its inhibition effectively mitigates reperfusion injury (Chen C. et al., 2021b). Another study on diabetes mellitus myocardial IRI (DIR) revealed that endoplasmic reticulum (ER) stress increased serum ACSL4 levels. Furthermore, DIR-induced ferroptosis amplified ER stress, exacerbating cardiomyocyte damage -an effect reversed by Ferrostatin-1. Similarly, renal IRI models in mice demonstrated ferroptosis accompanied by upregulated ACSL4 expression. ACSL4 levels progressively increased with extended ischemia or reperfusion duration. An intestinal IRI study further showed that pre-treatment with rosiglitazone or ACSL4-targeting siRNA prior to reperfusion inhibited ferroptosis and alleviated intestinal damage. This study identified Specificity Protein 1 (Sp1) as a key transcription factor promoting ACSL4 expression via binding to its promoter region, offering a novel regulatory target for preventing/treating intestinal IRI.

An epigenetics study on non-alcoholic steatohepatitis (NASH) found hypomethylation of the ACSL4 gene in peripheral blood lymphocytes, leading to its overexpression and suggesting its potential as a biomarker for NASH-associated ferroptosis. Arsenic exposure, a risk factor for NASH, also induces hepatocyte ferroptosis in a dose-dependent manner correlating with ACSL4 levels. Inhibiting ACSL4 activity with rosiglitazone or knocking down its expression via siRNA reduced cellular 5-HETE levels, significantly ameliorating arsenic-induced ferroptosis and NASH (Duan et al., 2022). Additionally, ACSL4 is implicated in ulcerative colitis (UC), an inflammatory condition characterized by dysregulated immunity. In a dextran sulfate sodium (DSS)-induced UC mouse model, ACSL4 upregulation coincided with ferroptosis onset. Ferroptosis inhibitors (Ferrostatin-1, Liproxstatin-1) normalized ACSL4 levels and suppressed ferroptosis (Gao et al., 2023).

Thiazolidinediones (TZDs), such as Rosiglitazone, Pioglitazone, and 2,4-thiazolidinedione, are PPARγ agonists used clinically to enhance insulin sensitivity in type 2 diabetes. These agents hold promise to control ACSL4-mediated pathological ferroptosis (Doll et al., 2017).

In summary, ACSL4-dependent ferroptosis occurs both in tumors with high ACSL4 expression (e.g., sorafenib-treated hepatocellular carcinoma) and in pathological processes like IRI affecting other tissues (e.g., transplantation). Key research challenges include: (1) Enhancing the tumoricidal efficacy of ferroptosis induction while minimizing toxicity to normal tissues, and (2) Determining whether sufficient therapeutic windows exist for ACSL4 inhibitors or other anti-ferroptosis interventions in conditions like IRI. Future research must elucidate the dynamic regulatory networks governing ACSL4 in different tissues and develop context-specific targeting strategies to balance its lethal *versus* protective functions.

### Molecular mechanisms of ferroptosis evasion in therapy-resistant tumors

Tumor cells resist ferroptosis through multiple complex mechanisms. These resistance strategies not only facilitate tumor initiation and progression but also contribute to therapy resistance and tumor recurrence. Here we briefly outline four major strategies employed by tumor cells to counteract ferroptosis: metabolic reprogramming, regulation of organelles and signaling pathways, and adaptation to the tumor microenvironment, offering new perspectives for overcoming tumor drug resistance.

### Metabolic reprogramming

In terms of lipid metabolism, cancer stem cells (CSCs) reduce the sensitivity to lipid peroxidation by downregulating ACSL4 expression, thereby decreasing the abundance of PUFA-PLs in the cell membrane. For example, studies have revealed that tumor-repopulating cells rely on PCK2-mediated phospholipid remodeling to resist ferroptosis, while CSCs promote PCK2 ubiquitination and degradation through the STAT3/HERC6 pathway, maintaining membrane phospholipids in a ferroptosis-resistant state (Long et al., 2025). Another study highlighted that in lung cancer stem cells, CPT1A forms a positive feedback loop with c-Myc, which simultaneously activates the NRF2/GPX4 antioxidant system and downregulates ACSL4 to reduce oxidizable phospholipid content in the cell membrane, achieving dual resistance to ferroptosis (Ma L. et al., 2024b).

Regarding energy metabolism regulation, tumor cells undergo metabolic reprogramming under hypoxia driven by HIF-1α, enhancing glycolysis and promoting lactate production (Kang et al., 2025). Research from Nanjing Medical University demonstrated that the acidic microenvironment created by lactate significantly enhances tumor cells’ resistance to ferroptosis independently of the canonical SLC7A11 and FSP1 systems, a mechanism particularly prominent in solid tumors (Zhang et al., 2025). Additionally, tumor cells employ enzymatic modifications to counteract ferroptosis, such as upregulating iPLA2-β expression to hydrolyze pre-formed oxidized phospholipids (PL-OOH) and block lipid peroxidation propagation. Furthermore, tumor cells enhance glutamine metabolism and glutathione synthesis pathways to boost GPX4 activity, thereby strengthening their capacity to eliminate lipid peroxides.

### Small molecule compounds targeting ACSL4 and lipid peroxidation

As previously described, targeting any of the components of lipid peroxidation production can intervene in iron death and thus exert a modulatory effect on disease. Major targets include oxygen radicals, LOX, POR, LPCAT3 and ACSL4 (Table 3).

**TABLE 3:** Small molecule compounds targeting ACSL4 and lipid peroxidation.

Drug molecule name	Target	Mechanism
vitamin E	O-H	Oxygen radical capture
CoQ10	O-H	Oxygen radical capture
Trolox	O-H	Oxygen radical capture
Fer-1	N-H	Oxygen radical capture
Lip-1	N-H	Oxygen radical capture
THN	O-H	Oxygen radical capture
Idebenone	O-H	Oxygen radical capture
Curcumin	5-LOX	Oxygen radical capture
CAPE	5-LOX	Oxygen radical capture
Catehin	5-LOX、12-LOX	Oxygen radical capture
Baicalein	5-LOX、12-LOX	Oxygen radical capture, Antioxidant, inhibits iron chelation
Umbelliprenin	5-LOX、12-LOX	Antioxidant
BAY-X-1005	5-LOX	Inhibitory activator protein
PD 146176	5-LOX、12-LOX	Antioxidant
MK 886	5-LOX、12-LOX	Inhibitory activator protein
Zileuton	5-LOX	inhibits iron chelation
Atreleuton	5-LOX	inhibits iron chelation
danthraquinone	P450	electron transport chain
tannic acid	P450	electron transport chain
Rosiglitazone	ACSL4	Inhibits lipid peroxides accumulation
pioglitazone	ACSL4	Inhibits lipid peroxides accumulation
troglitazone	ACSL4	Inhibits lipid peroxides accumulation
Triacsin C	ACSL1、ACSL3、ACSL4	Inhibits lipid peroxides accumulation
mefloquine	LPCAT3	Inhibits lipid peroxides accumulation
Thiazolidinediones	LPCAT3	Inhibits lipid peroxides accumulation
Selaginella trichoclada	LPCAT3	Inhibits lipid peroxides accumulation
kaempferol	LPCAT3	Inhibits lipid peroxides accumulation

### RTA

Free radical-trapping antioxidants (RTA) are molecules capable of inhibiting free radical propagation reactions, primarily including two types: phenols and aromatic amines. These compounds, typically lipophilic antioxidants, prevent lipid peroxidation and free radical chain reactions by capturing free radicals.

Ferrostatin-1(Fer-1), an antioxidant containing aromatic alkylamine, is one of the earliest discovered compounds with specific inhibitory activity against ferroptosis. It eliminates oxygen free radicals by inserting into the hydrophobic phospholipid bilayer, thereby suppressing ferroptosis. The conjugated double bond on the benzene ring of Fer-1 is believed to be the primary functional group responsible for this action. In various ferroptosis models induced by different means, Fer-1 significantly downregulates ferroptosis-related markers and genes at the micromolar level, preventing ferroptosis in cells. In inflammatory or injury models of mouse organs such as the heart, liver, kidney, and gastrointestinal tract, Fer-1 can alleviate tissue damage and cell death caused by ferroptosis. However, due to the susceptibility of Fer-1’s ester bond to cleavage, resulting in inactive hydrolysis products, its physicochemical properties and metabolic stability are poor. Therefore, Fer-1 is currently only widely used as a classic ferroptosis inhibitor in the study of ferroptosis-related diseases. Future structural modifications of Fer-1 are needed to improve its metabolic stability and achieve higher plasma concentrations for potential clinical applications (Zhou et al., 2022; Kajarabille and Latunde-Dada, 2019).

Liproxstatin-1(Lip-1), a compound containing amide and sulfonamide substructures, acts similarly to Fer-1. Both directly capture oxygen free radicals, thereby inhibiting cellular lipid peroxidation reactions, reducing oxidative stress, and suppressing ferroptosis (Xie et al., 2024). Lip-1 exhibits better physicochemical properties than Fer-1, with improved stability and drug absorption and distribution in the body. It is also widely used as a tool compound in ferroptosis research (Lillo-Moya et al., 2021; Yang K. et al., 2022c).

Vitamin E, a compound containing chroman ring, is a major source of antioxidants in people’s diets. It also has the ability to directly capture oxygen free radicals, but its ferroptosis inhibitory capacity is inferior to Fer-1 and Lip-1. This may be because its reaction rate with oxygen free radicals in the phospholipid bilayer is significantly lower than that of Fer-1 and Lip-1. The O-H bond in vitamin E is weaker than the N-H bond in Fer-1 and Lip-1 (Abraham et al., 2019; Traber and Head, 2021).

CoQ10, a phenolic RTA, has been widely used in clinical settings as an effective antioxidant and free radical scavenger. As a component of the mitochondrial respiratory chain, it exists in the lipid bilayer of the inner mitochondrial membrane, playing a role in electron transfer and reducing lipid peroxidation reactions in the inner mitochondrial membrane. CoQ10 carries electrons to reduce NAD+ and NADP + to NADH and NADPH, supplementing the cell’s reducing power through alternative pathways and neutralizing lipid radical-mediated auto-oxidation. This process is independent of GPX4’s inhibitory effect on ferroptosis. CoQ10 supplementation maintains cell membrane fluidity, reduces oxidative stress levels, lowers MDA and 4-HNE levels in the body, protects phospholipid membranes, alleviates redox imbalances in the body, and inhibits ferroptosis, especially in mitochondria-rich tissues and organs (Hargreaves et al., 2020; Bersuker et al., 2019; Zheng and Conrad, 2020).

Idebenone is a fat-soluble compound derived from CoQ10. It acts on the mitochondrial energy metabolism chain within cells. Used in Alzheimer’s disease to improve energy metabolism and glucose utilization, it increases ATP production in brain cells and inhibits lipid peroxidation in brain cell mitochondria. It is also commonly used as an antioxidant drug to eliminate aging-causing free radicals in the body (Gueven et al., 2021).

Tetrahydronaphthyridinol (THN), currently recognized as the most reactive phenolic RTA, operates similarly to Fer-1 and Lip-1 but reacts 100 times faster in the phospholipid bilayer (Zilka et al., 2017).

### LOX

LOX, a non-heme iron-containing dioxygenase, stands as a pivotal enzyme in catalyzing PUFA. Its primary substrates are unsaturated fatty acids. LOX boasts three main structural features: the presence of non-heme iron ions, a hydrophobic pocket, and a carboxylic acid binding site. Among the LOX family, 15-LOX holds a distinct position, owing to its specificity in oxidizing arachidonic acid at the 15th carbon atom, whence its name derives. This positional specificity marks a key difference between 15-LOX and other LOX enzymes. The redox-active non-heme iron in the structure of 15-LOX consists of four histidines and an isoleucine from the C-terminus.

Curcumin, the primary component of turmeric, has been proven beneficial for the prevention and treatment of various inflammatory diseases. The biosynthesis of arachidonic acid-derived lipid mediators, closely linked to inflammation, occurs through COX- and LOX-dependent pathways. Curcumin effectively suppresses the release of arachidonic acid and its metabolites in RAW cells stimulated by LPS, possibly by reducing COX-2 expression and inhibiting the catalytic activity of 5-LOX (Gupta et al., 2013).

Caffeic acid phenethyl ester (CAPE), extracted from propolis, is a potent antioxidant. As one of the natural products that have attracted much attention in recent years, CAPE possesses various pharmacological effects such as anti-cancer, anti-inflammatory, and anti-oxidant properties. CAPE can inhibit the production of free radicals formed by the xanthine dehydrogenase/xanthine oxidase system *in vitro*, prevent the formation of lipid peroxidation, and suppress the activities of tyrosine kinase, cyclooxygenase, and lipoxygenase (Ley-Martínez et al., 2022).

Catechin, a natural polyphenolic compound, exhibits antioxidant efficacy through two main mechanisms: (1) directly scavenging ROS and acting as a chelating agent for metal ions, and (2) indirectly activating antioxidant enzymes by inducing the expression of antioxidant enzymes and inhibiting the expression of pro-oxidant enzymes. In a study on the effect of catechin on LOX activity, it was found that catechin can effectively inhibit the catalytic activity of LOX, thereby reducing the generation of lipid peroxides to suppress ferroptosis (Sunil et al., 2021).

Zileuton, a classically approved LOX5 inhibitor for clinical use, has primarily been employed in anti-inflammatory treatments in previous studies. Recent research has revealed its significant inhibitory effect on LOX-induced ferroptosis. In a glutamate-induced HT22 cytotoxicity model, the application of zileuton demonstrated protective effects against glutamate-induced oxidative damage in HT22 cells and reduced associated cell death. This cell death model was also notably suppressed by the ferroptosis inhibitor Fer-1. However, no synergistic protective effect was observed between Fer-1 and zileuton. This suggests that the LOX5 inhibitor zileuton and the ferroptosis inhibitor Fer-1 exert their protective effects on glutamate-induced cell damage through the same cascade of actions. This further validates that zileuton exerts neuroprotective effects and reduces neuronal ferroptosis by inhibiting LOX5, thereby inhibiting the progression of neurological diseases (Hu WM. et al., 2023b).

Baicalein, a polyphenolic compound extracted from the traditional Chinese medicine Huang Qin, exhibits excellent antioxidant properties and functions as a LOX12/15 inhibitor. Baicalein can prevent FeCl3-induced post-traumatic epilepsy (PTE) seizures by inhibiting ferroptosis. After treatment with either a ferroptosis inhibitor or baicalein, the expression of LOX12/15 in the PTE model was significantly reduced, accompanied by a decrease in the production of 4-HNE and lipid ROS. This suggests that the attenuation of ferroptosis is associated with the neuroprotective effects of baicalein. Tang Daolin and colleagues found that baicalein can inhibit the effects of erastin, reducing the production of ferrous ions, the consumption of glutathione, the degradation of GPX4, and the generation of lipid peroxidation. These studies lay the foundation for baicalein’s inhibition of ferroptosis and provide new directions and ideas for the future application of baicalein targeting ferroptosis (Li et al., 2012).

Umbelliferone (UMB), an isoprenylated coumarin, functions as an antioxidant *in vitro* and inhibits lipoxygenase, which controls inflammatory pathways, while exhibiting anti-inflammatory activity *in vivo*. Overexpression of LOX15 is a driving factor in the malignant progression of prostate cancer. Studies have found that UMB can significantly inhibit the activity of LOX15, thereby exerting an anticancer effect on prostate cancer cells (Shahraki et al., 2020).

Veliflapon (BAY-X-1005) is an orally active, selective 5-LOX activating protein inhibitor. It also acts as an inhibitor of leukotriene B4 and C4 synthesis. Its core mechanism lies in directly inhibiting the catalytic activity of LOX, disrupting the conversion of arachidonic acid into inflammatory mediators such as leukotrienes. This not only reduces the recruitment of these mediators in inflammatory responses but also, through its unique chemical structure, potentially binds to the active site of LOX, further impeding substrate binding and the catalytic process. Through these combined effects, BAY-X-1005 demonstrates potential efficacy in the treatment of allergic asthma and other inflammatory diseases (Hatzelmann et al., 1993).

PD146176 is a synthetic inhibitor of LOX15. Previous research has shown that it can reverse cognitive impairment and cerebral amyloidosis by activating neuronal autophagy. Recent studies indicate that PD146176 can also inhibit ferroptosis. The Angeli team found that PD146176 effectively suppresses ferroptosis induced by GPX4 deficiency in mouse embryonic fibroblasts. In an erastin-induced ferroptosis model of human fibrosarcoma cells, PD146176 also effectively inhibited ferroptosis. The inhibition of LOX15 by PD146176 also reversed the TBH-induced ferroptosis model in cortical neurons (Da-Costa-Rocha and Prieto, 2021).

ML351 is another synthetic organic compound. Researchers identified this drug through high-throughput screening as a novel chemical inhibitor of LOX12/15, which may serve as a potential drug for nerve damage. In a glutamate-induced HT22 cell death model, ML351 exerts neuroprotective effects, protecting HT22 cells from glutamic acid oxidative toxicity and reducing neuronal ferroptosis. Further research has detected a significant increase in 12-HETE (a lipid peroxidation product of 12LOX/15LOX) in a mouse model of cerebral ischemic injury, consistent with observations in HT22 cells treated with glutamate. ML351 can reverse these effects, and its selectivity for LOX12/LOX15 is 250 times higher than that of other LOX isoenzymes (ChenG et al., 2021).

MK886 is an effective and selective inhibitor of 5-lipoxygenase-activating protein (FLAP). It can directly prevent the activation of LOX, inhibit the conversion of arachidonic acid into leukotrienes, and thereby reduce inflammatory responses. Additionally, MK-886 can affect the stemness of tumor cells by regulating LOX activity and down-regulating the expression of genes related to tumor cell stemness (Liao et al., 2023).

Atreleutonye, also known as ABT-761, is currently undergoing clinical trials as a novel 5LOX inhibitor. In a randomized controlled trial comparing the use of Atreleutonye and a placebo among patients with recent acute coronary syndrome, it was found that the group using Atreleutonye significantly reduced the risk of coronary atherosclerosis. However, this result still needs to be confirmed in larger-scale studies (Sinha et al., 2019).

### POR

P450 oxidoreductase (POR) is a key enzyme involved in steroid and drug metabolism, participating in the electron transfer process between microsomal cytochrome P450, cytochrome b5, squalene monooxygenase, and heme oxygenase. Recent studies have shown that POR activity is closely related to lipid peroxidation, and POR can act as a potential target for ferroptosis-inhibiting drugs.

Danthraquinone, a member of the anthraquinone compound family, possesses various biological activities such as anti-inflammatory, antibacterial, and antitumor effects. Studies have revealed that the specific chemical structures of Danthraquinone, including its aromatic rings and carbonyl groups, interact with the amino acid residues at the active site of the POR enzyme. This interaction blocks the binding of substrates to the enzyme and reduces its catalytic efficiency (Yang D. et al., 2022b).

Tannic acid (TA), a naturally occurring plant polyphenol, has been proven to reduce the mutagenicity and/or carcinogenicity of several amine derivatives and polycyclic aromatic hydrocarbons in rodents. Research has indicated that TA can decrease the level of CYP2E1 in animal livers and inhibit liver NQO1, and this effect is dose dependent. In an experiment evaluating the impact of TA on POR-catalyzed oxidation in human and rat liver microsomes, it was found that TA exhibits a non-selective effect on various enzymes, with the strongest inhibitory effect on CYP1A2. Additionally, the study revealed that TA also inhibits NADPH-POR reductase, which may partially explain its inhibitory effect on POR (Krajka-Kuźniak and Baer-Dubowska, 2003).

### ACSL4

As previously stated, ACSL4 directly participates in regulating intracellular PUFA to affect ferroptosis sensitivity by catalyzing the formation of AA-CoA/AdA-CoA from AA/AdA.

Rosiglitazone, as an agonist of peroxisome proliferator-activated receptor gamma (PPARγ), exerts its hypoglycemic effect by increasing the body’s sensitivity to insulin and improving cells’ utilization of glucose. Recent studies have shown that the use of rosiglitazone can inhibit ischemia/hypoxia-induced ACSL4 activation both *in vitro* and *in vivo*, thereby reducing ischemia/reperfusion-induced intestinal injury (Wang Y. et al., 2022b).

Pioglitazone and rosiglitazone share similar mechanisms of action. In a surgically induced knee osteoarthritis model, activating the PPARγ receptor can attenuate osteoarthritis progression and inhibit ferroptosis in chondrocytes. The administration of pioglitazone has been found to suppress the expression of the ferroptosis marker ACSL4 while restoring Pink1/Parkin-dependent mitochondrial autophagy and improving mitochondrial function (Kung et al., 2022).

Research has also revealed that irradiation (IR) significantly increases the expression level of the ferroptosis-promoting gene ACSL4 in mouse intestinal tissues, but this phenomenon is not observed in liver or lung tissues. Intervention with troglitazone reduces ACSL4 expression, mitigates lipid peroxidation in the intestine, and prevents IR-induced intestinal tissue damage.

### LPCAT3

AA-CoA and AdA-CoA undergo esterification under the action of LPCAT3 to produce phosphatidylethanolamine fatty chains, forming the precursors of ferroptosis execution molecules, namely, PE-AA and PE-AdA.

Ferroptosis plays a crucial role in enhancing PD-1 immunotherapy. Studies have found that Mef can induce ferroptosis to inhibit melanoma proliferation. Specifically, Mef upregulates the expression of LPCAT3 by promoting interferon (IFN)-γ-induced STAT1-IRF1 signal transduction, thus sensitizing melanoma and lung cancer to PD-1 immunotherapy (Tao et al., 2024).

This study investigated the anti-ferroptosis effect of suberosin (SBR, an isoprenyl coumarin) in diabetic rats and evaluated the efficacy of combining SBR with thiazolidinedione (TZ) in reducing TZ-induced cardiomyopathy. The results showed that after 4 weeks of TZ treatment alone, the cardiac output, stroke volume, left ventricular systolic and diastolic pressures of diabetic rats were all exacerbated. TZ treatment caused ferroptosis and myocardial cell tissue disorder. The study found that optimizing volume overload can reduce cardiac hypertrophy and alleviate left ventricular dysfunction (Jiang et al., 2024c). Combining SBR with TZ can reduce ferroptosis biomarkers in heart tissue and serum, downregulate the mRNA expression of LOX, ACSL4, and LPCAT3, promote GPX4 activity, and upregulate the mRNA levels of AKT/PI3K/GSK3β. Additionally, the combination of SBR helps maintain the normal tissue structure of cardiomyocytes. Therefore, SBR may be an effective complementary drug that can be used with TZ for diabetic patients, but this requires further clinical trials to confirm (IqbaL et al., 2023).

Multiple flavonoids were isolated from the 70% EtOH extract of Selaginella trichoclada. These compounds exhibited moderate toxicity towards human breast cancer MCF-7 cells, with compounds 2 and 8 showing stronger toxicity. Further investigation revealed that compound 8 could induce ferroptosis in MCF-7 cells by downregulating the expression of ferroptosis-related genes such as ACSL4 and could combat breast cancer drug resistance by inhibiting ACSL4 protein expression. This suggests its potential as a chemotherapeutic drug, but further validation is needed (Xie et al., 2022b).

Kaempferol (KP) possesses various biological effects including anti-inflammatory, antioxidant, anti-aging, and cardiovascular protective properties. Studies have explored the therapeutic mechanism of KP in the treatment of non-alcoholic steatohepatitis (NASH) through *in vivo* and *in vitro* experiments. The results demonstrated that KP significantly reduced the expression of LXRα, LPCAT3, and endoplasmic reticulum stress (ERS)-related factors in both NASH mouse models and steatotic cell models. This led to a decrease in hepatic steatosis and inflammation, indicating that KP improves ERS by lowering the expression levels of LXRα and LPCAT3, thereby exhibiting a potential therapeutic effect on NASH (Xiang et al., 2021).

### Prospects and perspectives

In summary, ACSL4 can promote ferroptosis in cancer cells through the esterification of AA and AdA. Current research has indicated that ACSL4 can serve as a biomarker for predicting the sensitivity of sorafenib treatment in liver cancer patients. Given the high expression of ACSL4 in various cancers, the specific activation of ferroptosis may represent an ideal anti-cancer strategy. However, one question remains: whether ferroptosis inducers like sorafenib can specifically target clinical tumor cells with high ACSL4 expression while sparing non-cancerous tissues with physiological ACSL4 expression. ACSL4 has been widely reported as a biomarker for ferroptosis sensitivity in numerous studies. It can promote the occurrence of ferroptosis, which not only kills tumor cells but also suggests a role for ACSL4 in promoting ferroptosis-induced diseases. ACSL4’s role in promoting ferroptosis primarily stems from its critical involvement in the metabolism of AA and AdA, as well as lipid peroxidation. Differences in fatty acid distribution across various tissues and tumors, coupled with variations in ACSL4 and other related lipid metabolism enzymes, contribute to significant disparities in ferroptosis sensitivity among different tissues and tumors. This provides valuable clues for exploiting ferroptosis to inhibit tumor cells. Currently, there is no simple and feasible biomarker for lipid peroxidation. However, the basal expression of ACSL4 and changes in its expression during disease progression could potentially serve as clinically valuable biomarkers. The analysis of resected or sampled tumor tissues may enable precise ferroptosis-based tumor therapy, although this hypothesis remains to be further validated.

Future clinical research should conduct multi-center, large-scale prospective cohort studies to systematically analyze serum ACSL4 expression levels in patients responsive or unresponsive to ferroptosis inducers. This aims to validate the reliability of serum ACSL4 as a biomarker for predicting sensitivity to ferroptosis-targeted therapies. Concurrently, a dynamic monitoring and individualized treatment system based on ACSL4 expression patterns should be established, providing a scientific foundation for the clinical translation of ferroptosis-based anticancer strategies.
